# Risk of Major Congenital Malformations and Exposure to Antiseizure Medication Monotherapy

**DOI:** 10.1001/jamaneurol.2024.0258

**Published:** 2024-03-18

**Authors:** Dina Battino, Torbjörn Tomson, Erminio Bonizzoni, John Craig, Emilio Perucca, Anne Sabers, Sanjeev Thomas, Silje Alvestad, Piero Perucca, Frank Vajda

**Affiliations:** 1 Fondazione IRCCS Istituto Neurologico Carlo Besta, Milan, Italy; 2 Department of Clinical Neuroscience, Karolinska Institutet, Stockholm, Sweden; 3 Department of Neurology, Karolinska University Hospital, Stockholm, Sweden; 4 RIDE2Med Foundation, Milan, Italy; 5 Belfast Health and Social Care Trust, Belfast, United Kingdom; 6 Department of Medicine (Austin Health), University of Melbourne, Melbourne, Victoria, Australia; 7 Department of Neuroscience, Monash University, Melbourne, Victoria, Australia; 8 University Hospital Rigshospitalet, Copenhagen, Denmark; 9 Institute for Communicative and Cognitive Neurology, Trivandrum, India; 10 National Centre for Epilepsy, Oslo University Hospital, Oslo, Norway; 11 Department of Clinical Medicine, University of Bergen, Bergen, Norway; 12 Bladin-Berkovic Comprehensive Epilepsy Program, Austin Health, Melbourne, Victoria, Australia; 13 Epilepsy Research Centre, Department of Medicine (Austin Health), The University of Melbourne, Melbourne, Victoria, Australia; 14 Department of Neuroscience, Central Clinical School, Monash University, Melbourne, Victoria, Australia; 15 Department of Neurology, The Royal Melbourne Hospital, Melbourne, Victoria, Australia; 16 Department of Neurology, Alfred Health, Melbourne, Victoria, Australia

## Abstract

**Importance:**

Women with epilepsy (WWE) require treatment with antiseizure medications (ASMs) during pregnancy, which may be associated with an increased risk of major congenital malformations (MCMs) in their offspring.

**Objective:**

To investigate the prevalence of MCMs after prenatal exposure to 8 commonly used ASM monotherapies and changes in MCM prevalence over time.

**Design, Setting, and Participants:**

This was a prospective, observational, longitudinal cohort study conducted from June 1999 to October 2022. Since 1999, physicians from more than 40 countries enrolled ASM-treated WWE before pregnancy outcome was known and followed up their offspring until 1 year after birth. Participants aged 14 to 55 years who were exposed to 8 of the most frequently used ASMs during pregnancy were included in this study. Data were analyzed from April to September 2023.

**Exposure:**

Maternal use of ASMs at conception.

**Main Outcomes and Measures:**

MCMs were assessed 1 year after birth by a committee blinded to type of exposure. Teratogenic outcomes across exposures were compared by random-effects logistic regression adjusting for potential confounders and prognostic factors.

**Results:**

A total of 10 121 prospective pregnancies exposed to ASM monotherapy met eligibility criteria. Of those, 9840 were exposed to the 8 most frequently used ASMs. The 9840 pregnancies occurred in 8483 women (mean [range] age, 30.1 [14.1-55.2] years). MCMs occurred in 153 of 1549 pregnancies for valproate (9.9%; 95% CI, 8.5%-11.5%), 9 of 142 for phenytoin (6.3%; 95% CI, 3.4%-11.6%), 21 of 338 for phenobarbital (6.2%; 95% CI, 4.1%-9.3%), 121 of 2255 for carbamazepine (5.4%; 95% CI, 4.5%-6.4%), 10 of 204 for topiramate (4.9%; 95% CI, 2.7%-8.8%), 110 of 3584 for lamotrigine (3.1%; 95% CI, 2.5%-3.7%), 13 of 443 for oxcarbazepine (2.9%; 95% CI, 1.7%-5.0%), and 33 of 1325 for levetiracetam (2.5%; 95% CI, 1.8%-3.5%). For valproate, phenobarbital, and carbamazepine, there was a significant increase in the prevalence of MCMs associated with increasing dose of the ASM. Overall prevalence of MCMs decreased from 6.1% (153 of 2505) during the period 1998 to 2004 to 3.7% (76 of 2054) during the period 2015 to 2022. This decrease over time was significant in univariable logistic analysis but not after adjustment for changes in ASM exposure pattern.

**Conclusions and Relevance:**

Of all ASMs with meaningful data, the lowest prevalence of MCMs was observed in offspring exposed to levetiracetam, oxcarbazepine, and lamotrigine. Prevalence of MCMs was higher with phenytoin, valproate, carbamazepine, and phenobarbital, and dose dependent for the latter 3 ASMs. The shift in exposure pattern over time with a declining exposure to valproate and carbamazepine and greater use of lamotrigine and levetiracetam was associated with a 39% decline in prevalence of MCMs, a finding that has major public health implications.

## Key Points

**Question:**

What is the comparative risk of major congenital malformations (MCMs) associated with antiseizure medication (ASM) monotherapy in the offspring of mothers with epilepsy taking ASMs during pregnancy?

**Findings:**

In this cohort study including 9840 pregnancies, offspring exposed to levetiracetam, oxcarbazepine, and lamotrigine had the lowest prevalence of MCMs, compared with other ASMs. An increased risk of MCMs was associated with increasing doses of carbamazepine, phenobarbital, and valproate; the overall prevalence of MCMs decreased by 39% over time, in parallel with declining use of valproate and carbamazepine and greater use of levetiracetam and lamotrigine.

**Meaning:**

Results of this study suggest essential information for safer treatment selection in women of childbearing potential requiring ASM therapy.

## Teaser

This cohort study investigates the risk of major congenital malformations associated with antiseizure medication (ASM) monotherapy in the offspring of mothers taking ASMs during pregnancy.

## Introduction

The possibility of prenatal exposure to antiseizure medications (ASMs) leading to increased risk of congenital anomalies has been a concern for more than 5 decades.^1^ Because most women with epilepsy (WWE) need to continue ASM treatment during pregnancy,^2^ identifying the safest treatment options for these women is essential. This information could also be applicable to women of childbearing potential who are taking ASMs for nonepilepsy conditions such as psychiatric disorders, migraine, and neuropathic pain.^3,4^ The assessment of pregnancy outcomes after exposure to different ASMs faces many challenges. Ethical constraints preclude the conduction of randomized studies, leaving observational studies as the sole option. Enrollment of large cohorts is also required to handle the influence of confounding variables.

To address these challenges, independent research groups have established prospective registries to compare the risk of major congenital malformations (MCMs) after prenatal ASM exposure.^5^ These registries have identified elevated risks after exposure to valproate and possibly topiramate, whereas risks have been lower for lamotrigine and levetiracetam.^6,7,8,9,10,11^ Some registries have reported a dose-dependent risk for valproate,^6,9,10^ carbamazepine,^9,10^ phenobarbital,^9,10^ and lamotrigine.^6,9,10^

Studies based on national health databases have confirmed increased risks linked to valproate and topiramate.^12,13^ Although advantageous due to their large sample size and population-based nature, these databases do not provide information on important details collected in dedicated registries, such as validation and classification of maternal epilepsy diagnosis, seizure control, and precise ASM dosage. Moreover, recording of MCMs in health databases is not based on standardized assessments as in dedicated registries.

The larger epilepsy and pregnancy registries have not published significant updates lately. The latest publication from the International Registry of Antiepileptic Drugs and Pregnancy (EURAP), a large international registry spanning across more than 40 countries, dates back to 2018 and reports data up to 2016.^9^ Since that time, EURAP has enrolled numerous additional pregnancies. At the same time, changes in ASM prescription patterns have occurred, warranting an updated analysis.

This study provides an updated assessment of the comparative risk of MCMs associated with the 8 most frequently used ASM monotherapies. It also provides new data on dose-outcome associations, changes over time in overall occurrence of MCMs, and the degree to which these changes correlate with changes in ASM prescription patterns.

## Methods

### Study Design and Participants

The EURAP registry is a longitudinal, prospective cohort study established in 1999 and extended to 47 countries with more than 1500 collaborators. The EURAP methodology has been described in detail before.^9,14^ The study protocol was approved by the ethics committees of participating centers. We obtained informed consent from all the women enrolled. The current analysis included pregnant WWE exposed to ASMs at the time of conception and enrolled within the 16th week of gestation, where fetal outcome had not yet been determined. We excluded pregnancies in women without epilepsy, pregnancies for which physicians did not submit reports within specified deadlines, and those for which follow-up was not yet completed at the current census. We also excluded pregnancies in which ASMs were switched or withdrawn during the first trimester, those exposed to ASM polytherapy or other known teratogenic drugs, and those with comorbidities associated with teratogenic risks (eTable 1 in Supplement 1). Spontaneous abortions, abortions induced for reasons unrelated to fetal abnormalities, pregnancies where fetal outcome could not be determined, and pregnancies leading to offspring with genetic or chromosomal abnormalities were also excluded (eTable 2 in Supplement 1). This study followed the Strengthening the Reporting of Observational Studies in Epidemiology (STROBE) reporting guidelines.

Race and ethnicity were ascertained by reporting physicians across participating countries and categorized in the case report form as Aboriginal, Asian, Black, North African, multiracial, Pacific Islander, White, and other or unknown. However, because some categories had minimal representation, this information was recategorized in the database into 3 main categories: Asian, White, and other, which included all other race categories listed in the case report form.

### Procedures

We collected information on demographics, epilepsy classification, seizure frequency, comorbidities, family history of MCMs, drug therapy, and other risk factors. Follow-up data were acquired after each trimester, at delivery, and 1 year after delivery. Reporting physicians were responsible for collecting the data, which were transmitted online to the central registry in Milan, Italy. Abnormalities in the offspring were recorded descriptively by reporting physicians. A committee blinded to type of exposure assessed and categorized these abnormalities. When necessary, the committee solicited additional information from the reporting physicians.

### Outcomes

Our primary aim was to compare the association of MCMs with the 8 most commonly used monotherapies (carbamazepine, lamotrigine, levetiracetam, oxcarbazepine, phenobarbital, phenytoin, topiramate, and valproate) assessed 1 year after birth in offspring exposed to these ASMs in utero and to compare risks across different dose ranges whenever a dose-dependent pattern emerged. We categorized pregnancies based on type and dosage of ASM at the time of conception. Seizures were categorized as either tonic-clonic (including focal to bilateral tonic-clonic or generalized tonic-clonic) or other types. Epilepsies were classified according to International League Against Epilepsy criteria.^15^ MCMs referred to structural abnormalities with medical, surgical, cosmetic, or functional significance classified according to the European Concerted Action on Congenital Anomalies and Twins (EUROCAT) 2005 criteria.^16^ The covariates assessed were selected a priori based on their potential relevance, drawing from our previous investigation of pregnancy outcomes.^9^ Family history of MCMs pertained to the proband’s parents, excluding cerebral malformations causally linked to maternal epilepsy. Folate supplementation was considered appropriate if initiated at least 3 months before conception and continued throughout the first trimester at a dose of 0.4 mg per day or greater.

### Statistical Analysis

According to the empirical rule of thumb that the ratio between the number of events and the number of explanatory variables should be 10 or greater, a sample size of 10 000 observations was deemed adequate to minimize the risk of overfitting in a multivariable logistic model, assuming a 5% frequency of teratogenic events in the pooled population and a selected number of plausible predictors no larger than 50. To calculate frequencies of MCMs, the numerator was the sum of all live births plus pregnancy losses with confirmed MCMs, whereas the denominator included live births, pregnancies terminated electively for suspected MCMs, and perinatal deaths. Dose categories were identified by a machine-based algorithm developed to overcome the limits of choosing dose intervals subjectively. The approach consists in splitting each treatment into 3 dose categories by identifying the combination of cutoffs that optimally separates the 3 dose intervals (more than 3 dose categories would add substantial complexity, with doubtful added gain). The process involves testing iteratively by logistic regression all possible cutoff pairs and selecting the combination with the largest C index among those showing a positive trend. If no positive correlation is detected, either because a dose-response association is absent or too weak, or the sample size is inadequate, a further attempt is made by testing 2 dose categories only. Of note, any identified association with only 2 dose categories is less robust and requires confirmation. The Cochrane-Armitage test was used to statistically test a dose-response association across the identified categories. A random-effects logistic model was used to compare treatments after adjusting for potential confounders or prognostic factors and to assess the effect of these factors on outcome. Logistic regression was done by fitting data through a generalized linear mixed model parameterized with logit-link function, binomial distribution, and inclusion of mother-level random effects to adjust for the presence of clustered data (women with more than 1 pregnancy). Results are reported as odds ratios (ORs) with 2-tailed 95% CIs. By setting the type II error at the same severity level as the type I error, multiplicity generated by the 55 comparisons was handled by controlling the false discovery rate through the Benjamini and Hochberg adaptive linear step-up approach^17,18^ as an alternative to classic Bonferroni type adjustments.^19^ To analyze changes in prevalence of MCMs over time, pregnancies were categorized based on 4 time periods of conception (1998-2004, 2005-2009, 2010-2014, and 2015-2022), which were defined a priori to ensure reasonably balanced numbers of pregnancies. The multivariable logistic model, which included time periods as independent variables, provided estimates of ORs for comparisons of time periods adjusted for other covariates, including changes in ASM prescription pattern (ASM type and dose category). Partially adjusted and unadjusted ORs were obtained by rerunning the random-effects logistic model parameterized with only the non-ASM covariates (partially adjusted) and time period as the only covariate (unadjusted) respectively. Statistical significance was set at a 2-sided *P* value <.05. Data were analyzed from April to September 2023 using SAS, version 9.4 (SAS Institute).

## Results

Of the 28 553 pregnancies entered into the database between June 20, 1999, and October 25, 2022, 10 121 exposed to ASM monotherapy and with complete follow-up data met criteria for inclusion (eFigure 1 in Supplement 1). Of these, 9840 had been exposed to the 8 most frequently used ASMs and were included in the primary analysis (Table 1). Of these, 1108 (11.3%) were pregnancies also entered in other collaborating registries (eTable 3 in Supplement 1). The 9840 pregnancies included in the primary analysis resulted in 9748 live births, 48 perinatal deaths (after 24 weeks of gestation and before 7 completed days), and 44 elective abortions for fetal abnormalities. A total of 1144 women contributed 2 pregnancies, 97 women contributed 3 pregnancies, 5 women contributed 4 pregnancies, and 1 woman contributed 5 pregnancies. Each of the 146 twin pregnancies and the 1 triplet pregnancy were considered separate pregnancies. Thus, the 9840 pregnancies occurred in 8483 women (mean [range] age, 30.1 [14.1-55.2] years). These women self-identified with the following race and ethnicity categories: 797 Asian (9.4%), 7365 White (86.8%), and 309 other (3.6%). For 12 women (0.1%), information on racial group was unavailable.

**Table 1.** Demographic and Clinical Data of the Study Cohort (N = 9840)

Characteristic	Mean (range)
Maternal age at time of conception, y	30.1 (14.1-55.2)
Duration of pregnancy at time of enrollment, wk	8 (1-16)
Parental history of major congenital malformations, No. (%)	
Negative	9669 (98.3)
Positive	111 (1.1)
Information missing	60 (0.6)
Geographical region, No. (%)	
North and South America	105 (1.1)
Europe	8460 (86.0)
Eastern Mediterranean	28 (0.3)
Southeast Asia	410 (4.2)
Western Pacific	837 (8.5)
Parity, No. (%)	
0	5847 (59.4)
1	3225 (32.8)
2	608 (6.2)
≥3	158 (1.6)
Information missing	2 (0.0)
Type of epilepsy, No. (%)	
Idiopathic generalized epilepsy	3959 (40.2)
Focal epilepsy	4865 (49.4)
Epilepsy of unknown type	1016 (10.3)
Tonic-clonic seizures during first trimester, No. (%)	
No	9182 (93.3)
Yes	637 (6.5)
Information missing	21 (0.2)
Educational level of the father, No. (%)^a^	
Low	1319 (13.4)
Medium or high	7811 (79.4)
Information missing	710 (7.2)
Educational level of the mother, No. (%)^a^	
Low	1180 (12.0)
Medium or high	8175 (83.1)
Information missing	485 (4.9)
Folic acid intake, No. (%)	
Appropriate	3631 (36.9)
Inappropriate	6136 (62.4)
Information missing	73 (0.7)
Offspring sex, No. (%)	
Female	4751 (48.3)
Male	5030 (51.1)
Information missing	59 (0.6)

^a^ Parental educational level was categorized into low (≤9 years of education) or medium or high (>9 years of education).

The prevalence of MCMs in the assessed offspring was 9.9% (153 of 1549 pregnancies; 95% CI, 8.5%-11.5%) for valproate, 6.3% (9 of 142 pregnancies; 95% CI, 3.4%-11.6%) for phenytoin, 6.2% (21 of 338 pregnancies; 95% CI, 4.1%-9.3%) for phenobarbital, 5.4% (121 of 2255 pregnancies; 95% CI, 4.5%-6.4%) for carbamazepine, 4.9% (10 of 204 pregnancies; 95% CI, 2.7%-8.8%) for topiramate, 3.1% (110 of 3584 pregnancies; 95% CI, 2.5%-3.7%) for lamotrigine, 2.9% (13 of 443 pregnancies; 95% CI, 1.7%-5.0%) for oxcarbazepine, and 2.5% (33 of 1325 pregnancies; 95% CI, 1.8%-3.5%) for levetiracetam (Table 2). For carbamazepine, phenobarbital and valproate, there was a significant increase in the prevalence of MCMs associated with increasing dose of the ASM (Table 2). Outcomes in the 281 pregnancies exposed to less frequently used ASMs are reported in eTable 4 in Supplement 1, whereas MCM prevalence data among cases lost to follow-up after delivery are summarized in eTable 5 in Supplement 1.

**Table 2.** Prevalence of Major Congenital Malformations (MCMs) in Offspring Exposed Prenatally to Monotherapy With 1 of 8 Different Antiseizure Medications (ASMs)^a^

ASM treatment (dose range, mg/d)	No.		Prevalence of MCMs (95% CI), %	Dose- dependency *P* value
	Exposed pregnancies	Pregnancies with MCMs		
Carbamazepine (25-2400)	2255	121	5.4 (4.5-6.4)	NA
Lamotrigine (5-1300)	3584	110	3.1 (2.5-3.7)	
Levetiracetam (80-5000)	1325	33	2.5 (1.8-3.5)	
Oxcarbazepine (75-4500)	443	13	2.9 (1.7-5.0)	
Phenobarbital (15-300)	338	21	6.2 (4.1-9.3)	
Phenytoin (30-730)	142	9	6.3 (3.4-11.6)	
Topiramate (25-600)	204	10	4.9 (2.7-8.8)	
Valproate (100-3000)	1549	153	9.9 (8.5-11.5)	
Phenobarbital (≤60)	76	2	2.6 (0.3-9.2)	.047
Phenobarbital (>60-≤130)	197	12	6.1 (3.2-10.4)	
Phenobarbital (>130)	65	7	10.8 (4.4-20.9)	
Carbamazepine (≤700)	1506	70	4.6 (3.6-5.8)	.008
Carbamazepine (>700 -≤1000)	541	32	5.9 (4.1-8.2)	
Carbamazepine (>1000)	208	19	9.1 (5.6-13.9)	
Valproate (≤650)	715	43	6.0 (4.4-8.0)	<.001
Valproate (>650-≤1450)	711	79	11.1 (8.9-13.6)	
Valproate (>1450)	123	31	25.2 (17.8-33.8)	

^a^ Prevalence at different dose ranges is given for ASMs where a dose dependency was identified.

**Figure 1.**
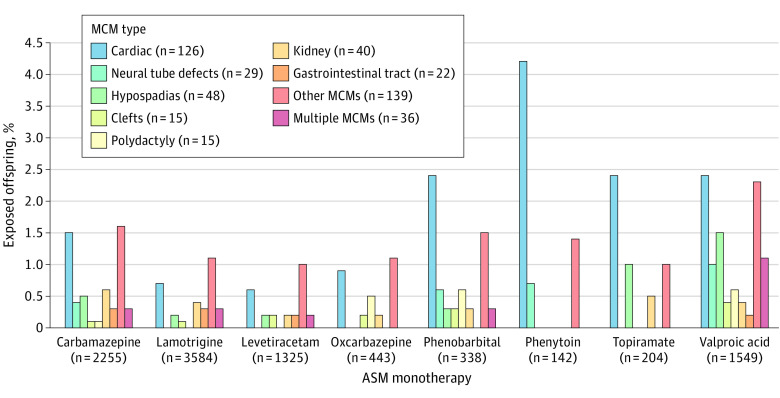
Prevalence of Different Categories of Major Congenital Malformations (MCMs) After Prenatal Exposure to Monotherapy With 8 Antiseizure Medications (ASMs) Prevalence (expressed as percentage of exposed offspring) of different categories of MCMs after prenatal exposure to monotherapy with the 8 most commonly used ASMs.

Cardiac malformations were the most frequent abnormality across ASMs, particularly after exposure to phenytoin, phenobarbital, topiramate, and valproate (Figure 1). Valproate exposure was associated with a wide range of MCMs, which also included hypospadias, neural tube defects, and multiple defects. There were no specific patterns associated with levetiracetam or lamotrigine (Figure 1).

Table 3 summarizes the results of the multivariable analysis where the teratogenic risk of individual ASMs was compared with that of the ASM associated with the lowest prevalence of MCMs (levetiracetam). Comparisons across other ASMs and risks associated with non-ASM covariates are also shown.

**Table 3.** Multivariable Analysis of Comparative Risk of Major Congenital Malformations in the Study Cohort

Comparison	OR (95% CI)	Adjusted *P* value^a^
**Drug comparisons with levetiracetam (≥80 mg/d to 5000 mg/d)**		
Carbamazepine (≤700 mg/d)	1.73 (1.10-2.72)	.02
Carbamazepine (>700 mg/d to ≤1000 mg/d)	2.23 (1.32-3.78)	.007
Carbamazepine (>1000 mg/d)	3.74 (2.01-6.96)	<.001
Phenobarbital (≤60 mg/d)	0.96 (0.22-4.14)	.96
Phenobarbital (>60 mg/d to ≤130 mg/d)	2.44 (1.21-4.92)	.02
Phenobarbital (>130 mg/d)	5.11 (2.09-12.45)	.001
Valproate (≤650 mg/d)	2.53 (1.54-4.16)	.001
Valproate (>650 mg/d to ≤1450 mg/d)	5.22 (3.32-8.20)	<.001
Valproate (>1450 mg/d)	14.27 (8.06-25.28)	<.001
Lamotrigine (≥5 mg/d to 1300 mg/d)	1.26 (0.84-1.89)	.26
Oxcarbazepine (≥75 mg/d to 4500 mg/d)	1.09 (0.56-2.13)	.80
Phenytoin (≥30 mg/d to 730 mg/d)	2.39 (1.08-5.31)	.04
Topiramate (≥25 mg/d to 600 mg/d)	2.02 (0.97-4.21)	.06
**Within-drug comparisons**		
Carbamazepine (>700 mg/d to ≤1000 mg/d vs ≤700 mg/d)	1.29 (0.83-1.99)	.26
Carbamazepine (>1000 mg/d vs ≤700 mg/d)	2.16 (1.26-3.71)	.01
Carbamazepine (>1000 mg/d vs >700 mg/d to ≤1000 mg/d)	1.68 (0.92-3.07)	.09
Phenobarbital (>60 mg/d to ≤130 mg/d vs ≤60 mg/d)	2.54 (0.55-11.74)	.23
Phenobarbital (>130 mg/d vs ≤60 mg/d)	5.32 (1.04-27.06)	.05
Phenobarbital (>130 mg/d vs >60 mg/d to ≤130 mg/d)	2.10 (0.77-5.68)	.15
Valproate (>650 mg/d to ≤1450 mg/d vs ≤650 mg/d)	2.06 (1.38-3.07)	.001
Valproate (>1450 mg/d vs ≤650 mg/d)	5.63 (3.31-9.58)	<.001
Valproate (>1450 mg/d vs >650 mg/d to ≤1450 mg/d)	2.74 (1.68-4.44)	<.001
**Other comparisons across ASMs**		
Carbamazepine (≤700 mg/d) vs lamotrigine (5-1300 mg/d)	1.37 (0.99-1.90)	.06
Oxcarbazepine (75-4500 mg/d) vs lamotrigine (5-1300 mg/d)	0.86 (0.48-1.56)	.63
Topiramate (25-600 mg/d) vs lamotrigine (5-1300 mg/d)	1.60 (0.82-3.13)	.17
Valproate (≤650 mg/d) vs lamotrigine (5-1300 mg/d)	2.01 (1.36-2.97)	.001
Carbamazepine (>700 mg/d to ≤1000 mg/d) vs lamotrigine (5-1300 mg/d)	1.77 (1.16-2.70)	.02
Phenobarbital (>60 mg/d to ≤130 mg/d) vs lamotrigine (5-1300 mg/d)	1.93 (1.03-3.62)	.049
Oxcarbazepine (75-4500 mg/d) vs carbamazepine (≤700 mg/d)	0.63 (0.34-1.16)	.14
Topiramate (25-600 mg/d) vs carbamazepine (≤700 mg/d)	1.16 (0.58-2.33)	.67
Valproate (≤650 mg/d) vs carbamazepine (≤700 mg/d)	1.46 (0.95-2.24)	.08
Other comparisons across ASMs		
Phenobarbital (>60 mg/d to ≤130 mg/d) vs carbamazepine (≤700 mg/d)	1.40 (0.74-2.68)	.30
Topiramate (25-600 mg/d) vs oxcarbazepine (75-4500 mg/d)	1.85 (0.79-4.35)	.16
Valproate (≤650 mg/d) vs oxcarbazepine (75-4500 mg/d)	2.32 (1.20-4.51)	.02
Carbamazepine (>700 mg/d to ≤1000 mg/d) vs oxcarbazepine (75-4500 mg/d)	2.04 (1.05-3.97)	.045
Phenobarbital (>60 mg/d to ≤130 mg/d) vs oxcarbazepine (75-4500 mg/d)	2.23 (0.99-5.06)	.06
Valproate (≤650 mg/d) vs topiramate (25-600 mg/d)	1.26 (0.61-2.59)	.54
Phenobarbital (>60 mg/d to ≤130 mg/d) vs topiramate (25-600 mg/d)	1.21 (0.50-2.90)	.67
Phenobarbital (>60 mg/d to ≤130 mg/d) vs valproate (≤650 mg/d)	0.96 (0.49-1.90)	.91
**Effect of non-ASM covariates**		
Parental history of major congenital malformations	3.43 (1.94-6.05)	<.001
Idiopathic generalized epilepsy vs focal epilepsy	0.81 (0.64-1.03)	.08
Epilepsy of unknown type vs focal epilepsy	0.81 (0.58-1.15)	.25
North and South America vs Europe	1.06 (0.45-2.51)	.89
Eastern Mediterranean vs Europe	1.16 (0.15-8.97)	.89
Southeast Asia vs Europe^b^	1.79 (1.17-2.73)	.01
Western Pacific vs Europe	1.05 (0.75-1.46)	.77
Maternal age at conception	1.01 (0.99-1.04)	.15
Tonic-clonic seizures during first trimester of pregnancy	0.89 (0.59-1.33)	.56
Folic acid (appropriate use vs no use or inappropriate use)	1.17 (0.96-1.44)	.12
Parity 1 vs 0	0.80 (0.64-0.99)	.049
Parity ≥2 vs 0	0.71 (0.48-1.06)	.10
Offspring sex	1.02 (0.84-1.23)	.84
Period 2005-2009 vs 1998-2004	0.97 (0.76-1.23)	.78
Period 2010-2014 vs 1998-2004	0.91 (0.69-1.21)	.51
Period 2015-2022 vs 1998-2004	1.00 (0.72-1.37)	.99

Abbreviations: ASM, antiseizure medication; OR, odds ratio.

^a^ Multiplicity generated by the 55 comparisons was handled by controlling false discovery rate, using the Benjamini and Hochberg adaptive linear step-up.

^b^ The higher prevalence of major congenital malformations is limited to cardiac malformations and is explained by the fact that the Kerala register routinely perform cardiac ultrasound examinations of all children in their cohort.

Phenytoin, the 2 highest dose categories of phenobarbital, and all dose categories of carbamazepine and valproate were associated with significantly higher risk of MCMs compared with levetiracetam. The low dose of valproate and the medium dose of carbamazepine and phenobarbital were associated with higher risk than lamotrigine. The low dose of valproate and the medium dose of carbamazepine were associated with higher risk than oxcarbazepine.

A parental history of MCMs was associated with a greater than 3-fold increase in risk. Neither epilepsy type nor occurrence of tonic-clonic seizures were associated with significantly increased risk of MCMs. The odds of MCMs were not reduced in pregnancies with periconceptional folate supplementation (Table 3).

**Figure 2.**
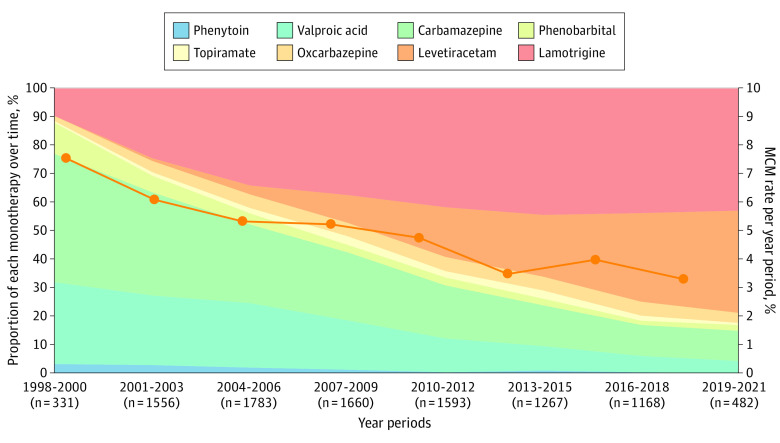
Proportion of Monotherapies per Year Groups (N = 9840) Changes in exposure to different antiseizure medication (ASM) monotherapies over time in the population included in the study and corresponding prevalence (%) of major congenital malformations (MCMs).

The prevalence of MCMs decreased over time from 6.1% (153 of 2505) during 1998 to 2004 to 5.1% (143 of 2825) during 2005 to 2009, 4.0% (98 of 2456) during 2010 to 2014, and 3.7% (76 of 2054) in 2015 to 2022. As shown in eTable 6 in Supplement 1, the decrease in MCM prevalence was significant at univariable logistic analysis (OR between first and last period, 0.59; 95% CI, 0.45-0.79) and at the multivariable logistic analysis, which includes only non-ASM covariates (OR between first and last period, 0.56; 95% CI, 0.42-0.75) but not after adjustment for changes in type of ASM exposure (Table 3). As shown in Figure 2, prescription patterns changed markedly over time, with a declining use of valproic acid and carbamazepine and greater use of lamotrigine and levetiracetam.

## Discussion

This updated analysis includes 2485 more prospective pregnancies (34%) than our 2018 analysis^9^ and adds 8 more years of follow-up for treatment changes and related risk implications.^14^ To our knowledge, this was the largest published cohort among epilepsy and pregnancy registries, permitting more precise estimation of the teratogenic risk associated with commonly used ASMs and other variables. The risk was lowest for levetiracetam, oxcarbazepine, and lamotrigine, and a dose dependency was identified for valproate, phenobarbital, and carbamazepine. In contrast with our earlier analysis, which included fewer lamotrigine-exposed pregnancies (2514 vs 3584 currently), we did not detect any dose dependency for lamotrigine.

Our multivariable analysis included 13 comparisons of the risk of MCMs between levetiracetam and other ASM exposures, 9 within-drug comparisons, 17 other comparisons across ASMs, and 16 comparisons with non-ASM covariates (Table 3). One important finding was that even at the lowest doses (≤650 mg per day) valproate was associated with a higher teratogenic risk compared with levetiracetam, lamotrigine, and oxcarbazepine—but not topiramate. The lowest doses of carbamazepine (≤700 mg per day) were associated with a higher risk than levetiracetam, whereas intermediate doses of carbamazepine (>700-1000 mg per day) were also associated with higher risk compared with lamotrigine and oxcarbazepine. Phenobarbital doses up to 60 mg per day were not associated with higher risk than levetiracetam, but the CIs were wide.

With respect to earlier studies, a network meta-analysis did not find increased risks with exposure to lamotrigine or levetiracetam^20^ nor did the Medicines and Healthcare Products Regulatory Agency UK Public Assessment Report.^21^ A population-based study using Nordic health databases found no increase in MCM risk with lamotrigine, levetiracetam, and oxcarbazepine but also no increased risk with carbamazepine.^12^ In contrast to our findings, the Nordic study found a dose-dependent increase in MCM risk with topiramate, similar to that of valproate.^12^ The latter study included ASM-exposed pregnancies regardless of indication, and dose estimates were derived from dispensing data rather than direct information from the treating physicians. The prevalence of MCMs after exposure to ASM monotherapies was further evaluated by a recent Cochrane review.^22^ Outcomes in cohort studies, such as EURAP and database-derived assessments, were evaluated separately. Of the approximately 25 000 pregnancies included in the Cochrane review, our previous report contributed 7355.^9^ Our current analysis of almost 10 000 pregnancies enabled 55 comparisons to be made between different ASM exposures and associations with non-ASM covariates. For comparisons made in both the current analysis and the Cochrane review (levetiracetam vs lamotrigine, levetiracetam vs oxcarbazepine, levetiracetam vs topiramate, oxcarbazepine vs lamotrigine, and lamotrigine vs topiramate) results were substantially similar.

The importance of accounting for the effect of covariates in multivariable analysis is demonstrated by our finding that parental history of MCMs was associated with a 3.4-fold increase in risk of MCMs in the offspring. This may imply a genetic predisposition to teratogenic effects of ASMs, as suggested by a higher risk of MCMs in the offspring of ASM-treated women who had a child with MCMs in a prior pregnancy while taking the same medication.^23,24^ Similar to earlier studies,^25,26,27,28,29^ we failed to identify a protective effect of folate supplementation against MCMs in ASM-exposed offspring. Periconceptional folate supplementation, however, has other benefits, including a decreased risk of preterm birth,^30^ improved cognitive development and verbal abilities in the offspring,^31,32,33^ and decreased risk of developing autistic traits postnatally.^34^ We did not analyze the precise doses of folate and, thus, could not contribute to the ongoing debate on optimal supplementation doses.^35,36^

A remarkable finding of our extended analysis, now spanning 24 years, is the continuous decline in overall prevalence of MCMs in offspring exposed to ASM monotherapies, from 6.1% in 1998 to 2004 to 3.7% (−39%) in 2015 to 2022. This decline is most likely related to a declining exposure to valproate and carbamazepine and an increased use of levetiracetam and lamotrigine (Figure 2). The fact that the decline in MCM rates was no longer significant after adjusting for changes in type of ASM exposure strongly suggests that these variables were causally related. Moreover, the categories of MCMs that declined most markedly (by at least 50% from first to last period) were neural tube defects, hypospadias, oral clefts, and polydactyly, some of which are associated to a greater extent with valproate and carbamazepine (Figure 1 and eFigure 2 in Supplement 1).

### Strengths and Limitations

Strengths of our study include a large sample size collected prospectively with rigorous methodology over many years, including 1-year follow-up of the offspring and blinded outcome assessment using standardized criteria. The assessment was not based on review of medical records but on direct reporting from the physician who was in charge of the clinical treatment of the women. Further strengths are the availability of accurate information on ASM doses at time of conception, seizure control, and other covariates, such as parental history of MCMs, all of which could be included in the multivariable analysis. Comparisons were based on data collected according to a standardized protocol, unlike the Cochrane review and other meta-analyses where comparisons included heterogeneous data from different sources.

Compared with studies based on national health databases, our cohort was not population based, which is an acknowledged limitation. EURAP pregnancies are likely to be enrolled by physicians with a special interest in epilepsy and pregnancy and, as such, possibly include women with more severe epilepsies but also more specialized management. Parental race was not included as a covariate in the analysis, but only a minority of pregnancies occurred in non-White women. A further limitation is the lack of a control group of pregnancies in women with untreated epilepsy. However, the question that EURAP aims to answer is which are the safest treatment options when ASM therapy needs to be continued during pregnancy. Although not directly comparable, the Cochrane review found that the prevalence of MCMs in the offspring of women without epilepsy was 2.1% in cohort studies and 3.3% in health record studies.^22^ These estimates are within the range found for levetiracetam, oxcarbazepine, and lamotrigine in our study.

## Conclusions

This cohort study provided essential information for physicians considering treatment options for WWE of childbearing potential, taking into consideration dose as well as type of ASM. Results suggest that exposure to levetiracetam, lamotrigine, and oxcarbazepine was associated with a low risk of MCMs and, in particular, a risk lower than that associated with the lowest dose category of valproate. We also identified a dose-dependent teratogenic risk associated with valproate, carbamazepine, and phenobarbital but not for lamotrigine. Although small numbers call for caution, the observation that a low dose of phenobarbital (≤60 mg per day) was not associated with an increased risk compared with any other ASM is particularly relevant for resource-limited settings, where phenobarbital may be the only affordable ASM. Most importantly, we observed major changes in the pattern of ASM prescription over time, resulting in a prominent decline in the overall prevalence of MCMs. Considering that there are up to 12 million WWE of childbearing potential worldwide and that additional women are exposed to ASMs for other indications,^4,37,38,39^ a 39% decline in the prevalence of MCMs in their offspring has major public health implications. Continued monitoring of teratogenic outcomes by registries and health databases is needed to assess the safety of prenatal exposure to newer generation ASMs. In addition to MCMs, other potential adverse consequences of prenatal ASM exposure, such as neurodevelopmental disorders,^32,37,40,41^ are equally important for treatment decisions and also require further investigation in future studies.

## Notes

Supplement 1. **eTable 1.** Exclusions for Comorbidities and Comedications Associated With Teratogenic Risks **eTable 2.** Exclusions for Chromosomal or Genetic Abnormalities in Offspring **eFigure 1.** Flowchart Describing Study Population and Selection Process **eTable 3.** Overlapping Data Among Other Pregnancies Registries **eTable 4.** Outcomes of Pregnancies Exposured to Monotherapy With Less Frequently Used ASMs **eTable 5.** MCM Prevalence Among the Cases Lost to Follow-Up After Delivery **eTable 6.** Tables With Time Trends in MCM Univariable and Multivariable Versions **eFigure 2.** MCM Types by Time Periods

Supplement 2. **Nonauthor Collaborators.** EURAP Collaborators.

Supplement 3.
Data Sharing Statement.
