# Supplementary material for: Risk of Major Congenital Malformations and Exposure to Antiseizure Medication Monotherapy
Source: JAMA Neurol. 2024 Mar 18;81(5):481–9. doi: 10.1001/jamaneurol.2024.0258 (PMC10949148; doi:10.1001/jamaneurol.2024.0258)
Supplement: Supplement 1. — eTable 1. Exclusions for Comorbidities and Comedications Associated With Teratogenic Risks eTable 2. Exclusions for Chromosomal or Genetic Abnormalities in Offspring eFigure 1. Flowchart Describing Study Population and Selection Process eTable 3. Overlapping Data Among Other Pregnancies Registries eTable 4. Outcomes of Pregnancies Exposured to Monotherapy With Less Frequently Used ASMs eTable 5. MCM Prevalence Among the Cases Lost to Follow-Up After Delivery eTable 6. Tables With Time Trends in MCM Univariable and Multivariable Versions eFigure 2. MCM Types by Time Periods [file jamaneurol-e240258-s001.pdf]

Supplementary Online Content

Battino D, Tomson T, Bonizzoni E, et al; EURAP Collaborators. Risk of major congenital malformations and exposure to antiseizure medication monotherapy. *JAMA Neurol*. Published online March 18, 2024. doi:10.1001/jamaneurol.2024.0258

eTable 1. Exclusions for Comorbidities and Comedications Associated with Teratogenic Risks.

eTable 2. Exclusions for Chromosomal or Genetic Abnormalities in Offspring.

eFigure 1. Flowchart Describing Study Population and Selection Process.

eTable 3. Overlapping Data Among Other Pregnancies Registries.

eTable 4. Outcomes of Pregnancies Exposed to Monotherapy with Less Frequently used ASMs.

eTable 5. MCM Prevalence Among the Cases Lost to Follow-Up After Delivery.

eTable 6. Tables with Time Trends in MCM Univariable and Multivariable Versions.

eFigure 2. MCM Types by Time Periods.

This supplementary material has been provided by the authors to give readers additional information about their work.

© 2024 Battino D et al. *JAMA Neurology.*

eTable 1, Supplement – Exclusions for comorbidities and comedications associated with teratogenic risks.

eTable 1A. Pregnancies excluded from the analysis because of other diseases with potential teratogenic outcome (N=325). The prevalence of malformations in the overall cohort was 4.6%, (95%CI 2.8% - 7.5%).

Diseases	Exposed pregnancies, n	Malformed Offspring	
		No. (%)	95%CI
Diabetes mellitus	112	5 (4.5)	(1.9 - 10.0)
Gestational diabetes mellitus	193	9 (4.7)	(2.5 - 8.6)
HIV, HCV infection	5	0 (0.0)	
Toxoplasmosis	9	1 (11.1)	(2.0 - 43.5)
Varicella (chickenpox)	5	0 (0.0)	
Hyperhomocysteinemia	1	0 (0.0)	
**Total**	**325**	**15 (4.6)**	**(2.8 - 7.5)**

© 2024 Battino D et al. *JAMA Neurology.*

eTable 1B. Pregnancies excluded from the analysis because of exposure to other potential teratogenic drugs (N=198). The prevalence of malformations in the overall cohort was 5.0%, (95%CI 2.8% - 9.0%).

Other drugs[a]	Exposed pregnancies, n	Malformed Offspring	
		No. (%)	95%CI
Aciclovir	1	0	
Alprazolam	2	0	
Amitriptyline	7	1 (14.3)	(2.6 - 51.3)
Aripiprazole	1	0	
Cannabis	3	0	
Citalopram	24	1 (4.2)	(0.7 - 20.2)
Clomipramine	2	0	
Cocaine	1	0	
Copaxone	1	1 (100.0)	(20.6 - 100.0)
Desvenlafaxine	2	0	
Duloxetine	2	0	
Escitalopram	15	1 (6.7)	(1.2 - 29.8)
Fluoxetine	10	1 (10.0)	(1.8 - 40.4)
Haloperidol	1	0	
Immunosuppressants	8	0	
Interferon	3	0	
Mirtazapine	4	0	
Modafinil	1	0	
Polytherapy *(more than one "other drug")*	28	1 (3.6)	(0.6 - 17.7)
Nortriptyline	1	0	
Opioid	2	0	
Opipramol	1	0	
Paroxetine	13	0	
Promethazine	1	0	
Quetiapine	5	0	
Sertraline	33	3 (9.1)	(3.1 - 23.6)
Sulpiride	1	0	
Trazodone	2	1 (50.0)	(9.4 - 90.5)
Valacyclovir	1	0	
Venlafaxine	20	0	
Vortioxetine	1	0	
Zolpidem	1	0	
**Total**	**198**	**10 (5.0)**	**(2.8 - 9.0)**

[a]Some of these drugs might currently not be considered teratogenic, but for the sake of consistency with previous EURAP publications, we have kept the same list for exclusions.

© 2024 Battino D et al. *JAMA Neurology.*

eTable 2, Supplement – Exclusions for chromosomal or genetic abnormalities in offspring (N=89).

Descriptions of chromosomal abnormalities & genetic syndromes	Pregnancies, n
Achondroplastic dwarfism (skeletal dysplasia)	1
Blepharophimosis-ptosis-epicanthus syndrome (BPES syndrome)	1
Chromosome abnormality (defective chromosomes 4 and 16); multiple malformations (spine, kidney, heart, diaphragm)	1
Chromosome abnormality, NOS	1
Chromosome abnormality, unspecified	1
Chromosome abnormality, unspecified; Congenital malformation of brain, unspecified	1
Chromosome replaced with ring or dicentric	1
Conductive and sensorineural hearing loss, inherited	1
Congenital cataract, inherited	1
Congenital glaucoma, inherited	1
Craniosynostosis, inherited	1
Di George's syndrome; congenital malformations of thymus; Tetralogy of Fallot	1
Down’s syndrome	27
Down’s syndrome, unspecified	2
Down’s syndrome, unspecified; Coarctation of aorta	1
Down’s syndrome; Atrial septal defect	1
Down’s syndrome; Congenital malformation of cardiac septum, unspecified	1

© 2024 Battino D et al. *JAMA Neurology.*

**Descriptions of chromosomal abnormalities & genetic syndromes**	**Pregnancies, n**
Down's syndrome; Congenital malformations of the heart, unspecified	1
Down's syndrome; Double inlet ventricle; Cystic hygroma	1
Down's syndrome; Double outlet right ventricle	1
Down's syndrome; Patent ductus arteriosus	1
Down's syndrome; Ventricular septal defect	1
Dravet syndrome	2
Edward's syndrome, unspecified	2
Edward's syndrome, unspecified; Congenital absence of upper arm and forearm with hand present; Longitudinal reduction defect of tibia; Reduction anomalies of cerebellum	1
Edward's syndrome, unspecified; Exomphalos; Medial cyst of face and neck	1
Freeman Sheldon Syndrom [*congenital malformation syndromes predominantly affecting facial appearance; osteochondrodysplasia with defects of growth of tubular bones and spine (Q77); arthrogryposis multiplex congenital*]	1
Goldenhar syndrome [*oculo-auriculo-vertebral syndrome [Hemifacial microsomia syndrome*]; Other congenital malformations of spine, not associated with scoliosis; microphthalmos	1
Incontinentia pigmenti, n.o.s	1
Male with sex chromosome mosaicism	1
Marfan's syndrome	2
Noonan's syndrome	1
Noonan's syndrome; congenital subaortic stenosis	1
Other deletions of part of a chromosome (15q13 microdeletion)	1
Other deletions of part of a chromosome (chromosome 15 microdeletion)	1

© 2024 Battino D et al. *JAMA Neurology.*

Descriptions of chromosomal abnormalities & genetic syndromes	Pregnancies, n
Other deletions of part of a chromosome (chromosome 2 deletion)	1
Other specified chromosome abnormalities (n.13, n.21, NOS); laryngomalacia	1
Other specified chromosome abnormalities (n.17, microduplication)	1
Other specified chromosome abnormalities (n.22, duplication); Trigonocephaly	1
Other specified chromosome abnormalities (n.22, NOS); Congenital malformations of the heart, unspecified	1
Patau's syndrome, unspecified	3
Patau's syndrome, unspecified; Congenital malformation of brain, unspecified	1
Triploidy and polyploidy	1
Triploidy and polyploidy; Holoprosencephaly	1
Trisomy 21, meiotic nondisjunction	1
Tuberous sclerosis	5
Turner's syndrome, unspecified	1
Turner's syndrome, unspecified; Coarctation of aorta	1
Unbalanced translocations and insertions; Atrioventricular septal defect; Ventricular septal defect	1
Wolff-Hirschorn syndrome; Atrial septal defect; Renal hypoplasia, unilateral	1
Wolff-Hirschorn syndrome; Hypospadias, unspecified	1

© 2024 Battino D et al. *JAMA Neurology.*

Descriptions of chromosomal abnormalities & genetic syndromes	Pregnancies, n
X-linked ichthyosis	1
Zellweger syndrome	1
**Total**	**89**

© 2024 Battino D et al. *JAMA Neurology.*

eFigure 1, Supplement - Flowchart describing study population and selection process.

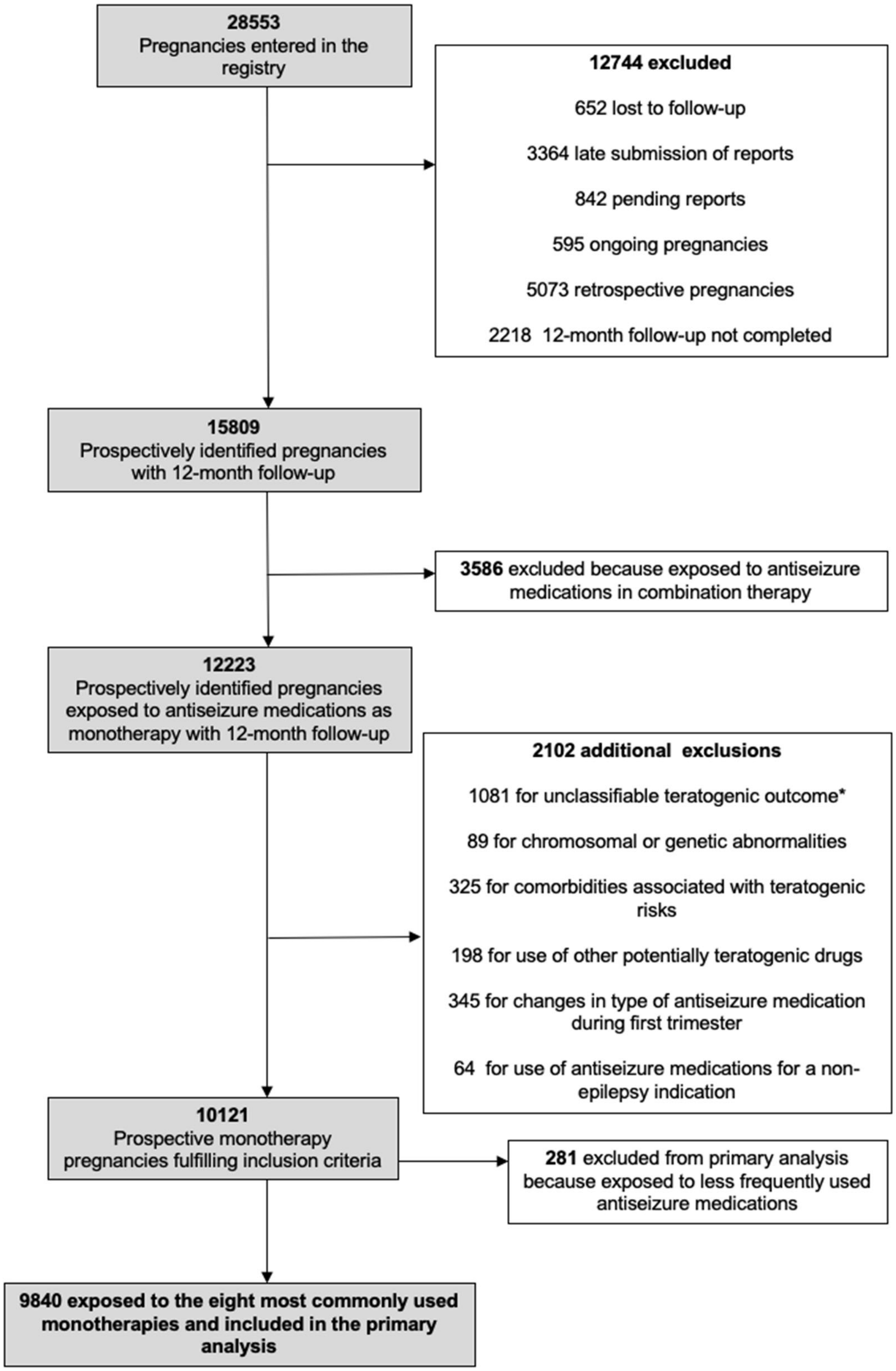

***Unclassifiable outcome (n=1081)**: Intrauterine deaths (n=898), induced abortions unrelated to foetal abnormalities (n=132) and unclear whether a major malformation was present (n=51).*

© 2024 Battino D et al. *JAMA Neurology.*

eTable 3, Supplement - Overlapping data among other pregnancies registries.

Pregnancies included in the analysis, highlighting number of cases also included in epilepsy and pregnancy registries from individual countries (N=9840).

Included cases by Country	N	%
Australia[a]	464	4.7
India[b]	410	4.2
United Kingdom[c]	234	2.4
**Total number of cases included in other registries**	**1108**	**11.3**
Other countries	8732	88.7
**Total number of cases included in the analysis**	**9840**	**100**

[a]Australian Pregnancy Register.

[b]Kerala Registry of Epilepsy and Pregnancy.

[c]UK and Ireland Epilepsy and Pregnancy Register.

© 2024 Battino D et al. *JAMA Neurology.*

eTable 4, Supplement - Outcomes of pregnancies exposed to monotherapy with less frequently used ASMs.

Number of prospective pregnancies exposed to 16 less frequently used ASMs as monotherapy and associated teratogenic outcomes (N=281). The prevalence of malformations in the overall cohort was 2.5%, (95%CI 1.2% - 5.0%).

ASM monotherapy	Exposed pregnancies, n	Malformed Offspring	
		No. (%)	95%CI
Acetazolamide	2	0 (0.0)	
Barbexaclone	23	0 (0.0)	
Brivaracetam	2	0 (0.0)	
Clobazam	19	1 (5.3)	(0.9 - 24.6)
Clonazepam	52	0 (0.0)	
Eslicarbazepine acetate	7	0 (0.0)	
Ethosuximide	18	0 (0.0)	
Felbamate	3	0 (0.0)	
Gabapentin	37	1 (2.7)	(0.5 - 13.8)
Lacosamide	31	0 (0.0)	
Methsuximide	1	0 (0.0)	
Pregabalin	7	0 (0.0)	
Primidone	44	3 (6.8)	(2.3 - 18.2)
Sulthiame	1	0 (0.0)	
Vigabatrin	5	1 (20.0)	(3.6 - 62.4)
Zonisamide	29	1 (3.4)	(0.6 - 17.2)
**Total**	**281**	**7 (2.5)**	**(1.2 - 5.0)**

© 2024 Battino D et al. *JAMA Neurology.*

eTable 5, Supplement - MCM Prevalence among the cases lost to follow-up after delivery.

MCM Prevalence among the cases lost to follow-up after delivery (N=1550).

Outcome	N	%
MCM	30	1.9
No Malformation	1520	98.1
**Total**	**1550**	**100**

© 2024 Battino D et al. *JAMA Neurology.*

eTable 6, Supplement –Tables with time trends in MCM univariable and multivariable versions.

eTable 6A. Frequency of MCMs associated with Periods and Univariable Logistic Analysis with Between Period Comparisons. (Unadjusted Estimates).

Period	Events	Sample	Crude Rate	Odds Ratio	Lower 95% CL	Upper 95% CL	P-Value
1998-2004	153	2505	6.10%	-	-	-	-
2005-2009	143	2825	5.10%	0.821	0.648	1.041	0.103
2010-2014	98	2456	4.00%	0.641	0.493	0.833	0.0009
2015-2022	76	2054	3.70%	0.592	0.446	0.787	0.0003

Note: Odds ratios and associated p-values are consistent with a decrease in the prevalence of crude MCM across time.

eTable 6B. Multivariable Logistic Analysis with only non-ASM as covariates.

Comparisons	Odds Ratio	Lower 95% CL	Upper 95% CL	P-Value
Parental history of major congenital malformations	3.367	1.931	5.869	< 0.0001
Idiopathic generalized epilepsy vs focal epilepsy	1.054	0.863	1.286	0.6059
Epilepsy of unknown type vs focal epilepsy	0.856	0.61	1.202	0.3694
Americas vs Europe	1.345	0.579	3.123	0.4907
Eastern Mediterranean vs Europe	1.053	0.139	7.958	0.9601
South-East Asia vs Europe	1.933	1.29	2.896	0.0014
Western Pacific vs Europe	1.105	0.798	1.528	0.5478

© 2024 Battino D et al. *JAMA Neurology.*

Comparisons	Odds Ratio	Lower 95% CL	Upper 95% CL	P-Value
Maternal age at conception	1.017	0.997	1.039	0.1027
Generalised tonic-clonic seizures during first trimester of pregnancy	0.866	0.579	1.294	0.4814
Folic acid (appropriate use vs no use or inappropriate use)	1.148	0.94	1.403	0.1756
Parity 1 vs 0	0.801	0.646	0.992	0.0421
Parity ≥ 2 vs 0	0.735	0.496	1.089	0.1248
Offspring sex	0.991	0.821	1.195	0.9224
Period 2005-2009 vs. Period 1998-2004	0.816	0.643	1.036	0.0946
Period 2010-2014 vs. Period 1998-2004	0.614	0.471	0.802	0.0003
Period 2015-2022 vs. Period 1998-2004	0.563	0.422	0.752	0.0001

Note: Odds ratios and associated p-values for the “between periods comparisons” are adjusted only for non-ASM covariates. Despite adjustment for non-ASM covariates, the “partially” adjusted estimates are still consistent with a decrease in the prevalence of MCM across time, just as for the unadjusted ones.This means that the decline in MCM rate is likely to be mainly or even fully ascribed to a different allocation (prevalence) of the ASMs in the four different periods. In fact, after adjusting also for changes in type of ASM exposure, the MCM decline was no longer detectable (Table 3).

© 2024 Battino D et al. *JAMA Neurology.*

eFigure 2, Supplement - MCM types by time periods.

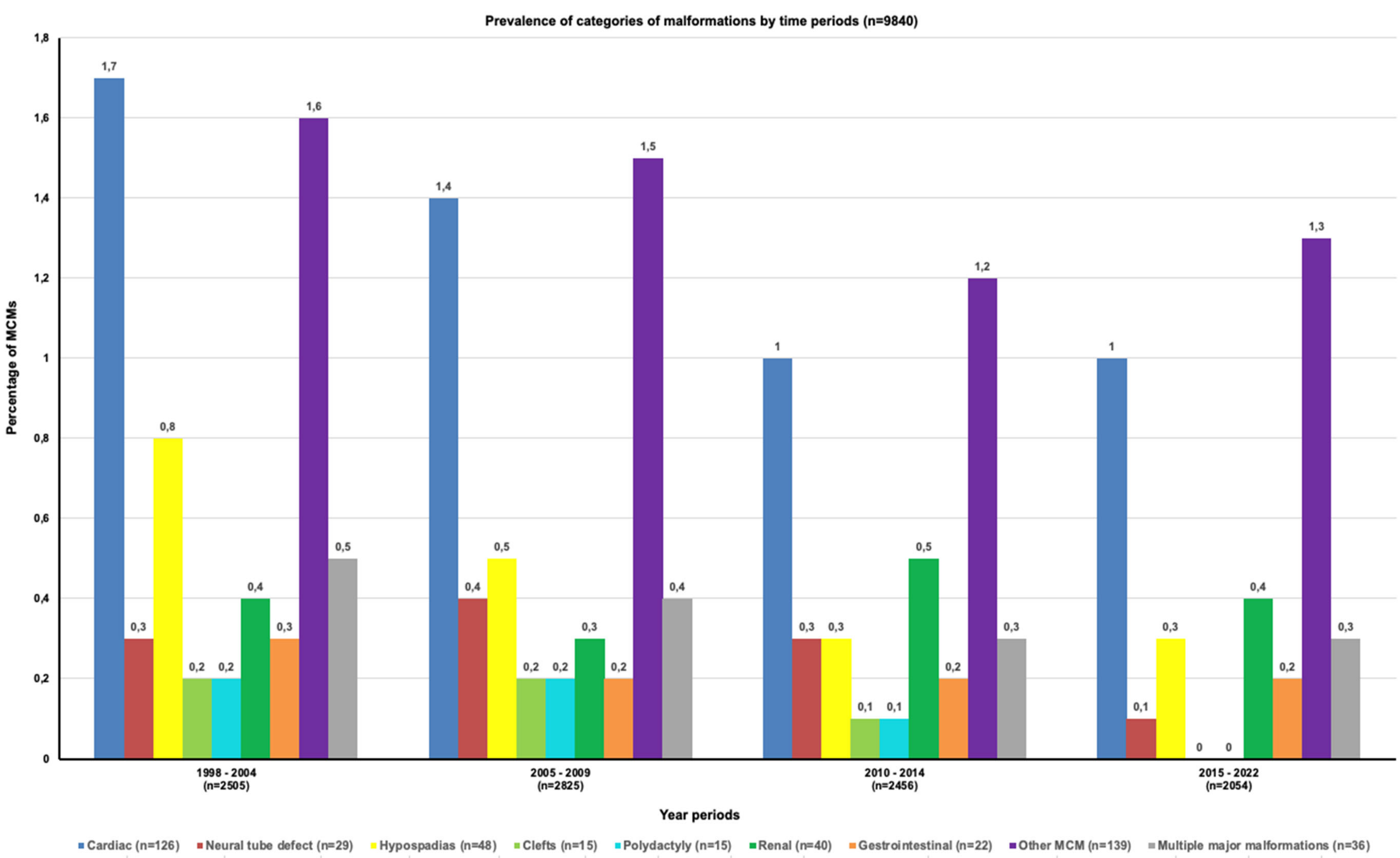

© 2024 Battino D et al. *JAMA Neurology.*

© 2024 Battino D et al. *JAMA Neurology.*
