# Supplementary material for: Risk of Major Congenital Malformations and Exposure to Antiseizure Medication Monotherapy
Source: JAMA Neurol. 2024 Mar 18;81(5):481–9. doi: 10.1001/jamaneurol.2024.0258 (PMC10949148; doi:10.1001/jamaneurol.2024.0258)
Supplement: Supplement 2. — Nonauthor Collaborators. EURAP Collaborators. [file jamaneurol-e240258-s002.pdf]

Supplemental Online Content: Nonauthor Collaborators
*First name, last name, and suffix (if applicable) are required and will appear in PubMed.

*Group Name(s): EURAP Collaborators

*First Name and Middle Initial(s)	*Last Name	*Suffix (eg, Jr, III)	Academic Degrees	Institution	Location (city, state/province, country)	Role or Contribution, eg, chair, principal investigator	Group (if more than 1 Group listed in the byline) and/or Subgroup (eg, Steering Committee)
Chiara	Pantaleoni		MD	Pediatric Neurosciences Department, Fondazione IRCCS Istituto Neurologico Carlo Besta	Milan, Italy	Outcome Assessment Committee	Outcome Assessment Committee
Claudia	Ciaccio		MD	Pediatric Neurosciences Department, Fondazione IRCCS Istituto Neurologico Carlo Besta	Milan, Italy	Outcome Assessment Committee	Outcome Assessment Committee
Silvia	Kochen		MD, PhD	National Council of Scientific and Technology Research (CONICET)	Buenos Aires, Argentina	National Coordinator	National Coordinator
Frank	Vajda		Professor,	Australian Pregnancy Register, Royal Melbourne Hospital	Parkville, Australia	National Coordinator	National Coordinator
Gerhard	Luef		Professor,	Department of Neurology, Epilepsy Unit, Medical University Innsbruck	Innsbruck, Austria	National Coordinator	National Coordinator
Alejandro	De Marinis		MD	Liga Chilena contra la Epilepsia	Santiago, Chile	National Coordinator	National Coordinator
Jana	Zarubova		MD	Department of Neurology, Second Faculty of Medicine, Charles University and Motol University Hospital	Prague, Czechia	National Coordinator	National Coordinator
Anne	Sabers		MD, DMSc	Rigshospitalet, Copenhagen University Hospital	Copenhagen, Denmark	National Coordinator	National Coordinator
Reetta	Kälviäinen		MD, PhD	Kuopio University Hospital	Kuopio, Finland	National Coordinator	National Coordinator
Sofia	Kasradze		Professor,	Caucasus International University	Tbilisi, Georgia	National Coordinator	National Coordinator
Bettina	Schmitz		Professor,	Vivantes Humboldt Hospital, Department of Neurology	Berlin, Germany	National Coordinator	National Coordinator
Sanjeev V.	Thomas		MD, PhD	Institute for communicative and cognitive neurology	Trivandrum, India	Central Project commission, National Coordinator	Central Project commission

Supplemental Online Content: Nonauthor Collaborators
*First name, last name, and suffix (if applicable) are required and will appear in PubMed.

***First Name and Middle Initial(s)**	***Last Name**	***Suffix (eg, Jr, III)**	Academic Degrees	Institution	Location (city, state/province, country)	Role or Contribution, eg, chair, principal investigator	Group (if more than 1 Group listed in the byline) and/or Subgroup (eg, Steering Committee)
Nasim	Tabrizi		MD, Associate professor of neurology, Epilepsy fellowship	Mazandaran University of medical sciences	Sari, Iran	National Coordinator	National Coordinator
Lilach	Goldstein		MD	Tel Aviv Medical Center	Tel Aviv, Israel	National Coordinator	National Coordinator
Barbara	Mostacci		MD, PhD	IRCCS Istituto delle Scienze Neurologiche di Bologna- Full Member of the ERN EpiCARE	Bologna, Italy	National Coordinator	National Coordinator
Hideyuki	Ohtani		MD	Shizuoka Institute of Epilepsy and Neurological Disorders	Shizuoka, Japan	National Coordinator	National Coordinator
Gordana	Kiteva-Trenchevska		Professor,	Medical Faculty, University "Ss Cyril and Methodius"	Skopje, Macedonia	National Coordinator	National Coordinator
Eugène	van Puijenbroek		Professor,	Netherlands Pharmacovigilance centre Lareb	s-Hertogenbosch, the Netherlands	National Coordinator	National Coordinator
Silje	Alvestad		MD, PhD	The National Centre for Epilepsy, Member of the ERN EpiCARE, Oslo University Hospital, Oslo, Norway; University of Bergen, Bergen	Oslo & Bergen, Norway	Central Project commission, National Coordinator	Central Project commission
Maja	Milovanović		MD, Assist	Institute of mental health & Faculty for Special Education and Rehabilitation, University of Belgrade	Belgrade, Serbia	National Coordinator	National Coordinator
Vladimír	Šafčák		MD	Department of Neurology, Air Force Military Hospital	Kosice, Slovakia	National Coordinator	National Coordinator
Meritxell	Martinez Ferri		MD	Hospital Universitari MútuaTerrasa	Barcelona, Spain	National Coordinator	National Coordinator

Supplemental Online Content: Nonauthor Collaborators
*First name, last name, and suffix (if applicable) are required and will appear in PubMed.

***First Name and Middle Initial(s)**	***Last Name**	***Suffix (eg, Jr, III)**	Academic Degrees	Institution	Location (city, state/province, country)	Role or Contribution, eg, chair, principal investigator	Group (if more than 1 Group listed in the byline) and/or Subgroup (eg, Steering Committee)
Torbjörn	Tomson		Professor,	Department of Clinical Neuroscience, Karolinska Institutet & Department of Neurology, Karolinska University Hospital	Stockholm, Sweden	Chair, Central project commission, National Coordinator	Central Project Commission
Elisabeth	Sellitto		MD, PhD	Swiss Epilepsy Center - Klinik Lengg	Zurich, Switzerland	National Coordinator	National Coordinator
Hsiang-Yu	Yu		MD	Neurology Department, Taipei Veterans General Hospital & National Yang Ming Chao Tung University	Taipei, Taiwan	National Coordinator	National Coordinator
Stephanie	Hödl		MD	Department of Neurology, Ghent University Hospital	Ghent, Belgium	Reporting Physician	Local recruiting site
Petr	Marusic		Professor,	Department of Neurology, Second Faculty of Medicine, Charles University and Motol University Hospital	Prague, Czechia	Reporting Physician	Local recruiting site
Renata	Listonova		MD	Outpatient Neurology Clinic, Falta s.r.o.	Náchod, Czechia	Reporting Physician	Local recruiting site
Hana	Krijtová		MD	Department of Neurology, Second Faculty of Medicine, Charles University and Motol University Hospital	Prague, Czechia	Reporting Physician	Local recruiting site
David	Franc		MD	Department of Neurology, University Hospital Olomouc	Olomouc, Czechia	Reporting Physician	Local recruiting site
Petr	Busek		MD, PhD, A	Outpatient Neurology Clinic, Medicon, Budejovicka	Prague, Czechia	Reporting Physician	Local recruiting site
Michaela	Kajšová		MD	Department of Neurology, Second Faculty of Medicine, Charles University and Motol University Hospital	Prague, Czechia	Reporting Physician	Local recruiting site
Noemi	Andersen		MD	Rigshospitalet, Copenhagen University Hospital	Glostrup, Denmark	Reporting Physician	Local recruiting site
Birthe	Pedersen		MD	The Danish Epilepsy Center, Filadelfia	Dianalund, Denmark	Reporting Physician	Local recruiting site

Supplemental Online Content: Nonauthor Collaborators
*First name, last name, and suffix (if applicable) are required and will appear in PubMed.

***First Name and Middle Initial(s)**	***Last Name**	***Suffix (eg, Jr, III)**	Academic Degrees	Institution	Location (city, state/province, country)	Role or Contribution, eg, chair, principal investigator	Group (if more than 1 Group listed in the byline) and/or Subgroup (eg, Steering Committee)
Katarzyna	Mieszczanek		MD	The Danish Epilepsy Center, Filadelfia	Dianalund, Denmark	Reporting Physician	Local recruiting site
Katarzyna	Cebula		MD	The Danish Epilepsy Center, Filadelfia	Dianalund, Denmark	Reporting Physician	Local recruiting site
Stefan	Juhl		MD	The Danish Epilepsy Center, Filadelfia	Dianalund, Denmark	Reporting Physician	Local recruiting site
Birgitte	Forsom Sondal		MD	Gødstrup Regional Hospital	Holstebro, Denmark	Reporting Physician	Local recruiting site
Karen	Nielsen		MD	Gødstrup Regional Hospital	Holstebro, Denmark	Reporting Physician	Local recruiting site
Tatiana V.	Danielsen		MD	University Hospital, Aalborg	Aalborg, Denmark	Reporting Physician	Local recruiting site
Elsebeth	Bruun Christiansen		MD	University Hospital, Aalborg	Aalborg, Denmark	Reporting Physician	Local recruiting site
Jakob	Christensen		Professor	Aarhus Universitetshospital, Palle Juul-Jensens Bouldevard 165	Aarhus N, Denmark	Reporting Physician	Local recruiting site
Ovidio	Solano Cabrera		MD	Instituto Salvadoreño del Seguro Social	San Salvador, El Salvador	Reporting Physician	Local recruiting site
Aleksei	Rakitin		MD, PhD	Neurology Clinic, Tartu University Hospital, Tartu, Estonia	Tartu, Estonia	Reporting Physician	Local recruiting site
Anne	Kirss		MD	Women’s Clinic of Tartu University Hospital	Tartu, Estonia	Reporting Physician	Local recruiting site
Anna M.	Saukkonen		MD	Pohjois-Karjala Central Hospital	Joensuu, Finland	Reporting Physician	Local recruiting site
Nino	Gogatishvili		MD, PhD, A	Caicasus International University	Tbilisi, Georgia	Reporting Physician	Local recruiting site
Dieter	Dennig		MD	Neurological community practice "Am Seelberg"	Stuttgart, Germany	Reporting Physician	Local recruiting site
Kerstin	Erdmann		MD	Neurological community practice "Am Seelberg"	Stuttgart, Germany	Reporting Physician	Local recruiting site
Christian	Dippon		MD	Neurological community practice "Am Seelberg"	Stuttgart, Germany	Reporting Physician	Local recruiting site
Bernhard	Steinhoff		Professor,	Centre of Epilepsy, Kehl Kork	Kehl Kork, Germany	Reporting Physician	Local recruiting site
Lisa M.	Langenbruch		MD	University of Münster, Department of Neurology	Münster, Germany	Reporting Physician	Local recruiting site
Holger	Lerche		Professor,	University of Tübingen, Department of Neurology	Tübingen, Germany	Reporting Physician	Local recruiting site
Anja	Herzer		MD	Centre of Neurology & Epilepsy Alsterdorf	Hamburg, Germany	Reporting Physician	Local recruiting site
Jan S.	Gerdes		MD	Centre of Neurology & Epilepsy Alsterdorf	Hamburg, Germany	Reporting Physician	Local recruiting site

Supplemental Online Content: Nonauthor Collaborators
*First name, last name, and suffix (if applicable) are required and will appear in PubMed.

***First Name and Middle Initial(s)**	***Last Name**	***Suffix (eg, Jr, III)**	Academic Degrees	Institution	Location (city, state/province, country)	Role or Contribution, eg, chair, principal investigator	Group (if more than 1 Group listed in the byline) and/or Subgroup (eg, Steering Committee)
Elisa K.	El-Allawy-Zielke		MD	Centre of Neurology & Epilepsy Alsterdorf	Hamburg, Germany	Reporting Physician	Local recruiting site
Hajo	Hamer		Professor,	University Friedrich-Alexander of Erlangen, University hospital, Department of Neurology and Epileptology	Erlangen, Germany	Reporting Physician	Local recruiting site
Malgorzata	Kalita		MD	Centre of Epilepsy Kleinwachau	Sachsen, Germany	Reporting Physician	Local recruiting site
Martin	Hirsch		MD	University of Freiburg, Department of Neurology	Freiburg, Germany	Reporting Physician	Local recruiting site
Stephan	Arnold		MD	Centre of Neurology, Private Practice "MVZ Nymphenburg"	München, Germany	Reporting Physician	Local recruiting site
Hans-Beatus	Straub		Professor,	Center for Epilepsy Berlin-Brandenburg: Königin Elisabeth Herzberge Hospital, Bernau	Berlin - Brandenburg, Germany	Reporting Physician	Local recruiting site
Rebekka	Lehmann		MD	Center for Epilepsy Berlin-Brandenburg: Königin Elisabeth Herzberge Hospital, Bernau	Berlin - Brandenburg, Germany	Reporting Physician	Local recruiting site
Christiane	Asenbauer		MD	Centre of Neurology, Private Practice, Dr. Christiane Asenbauer, Tübingen	Tübingen, Germany	Reporting Physician	Local recruiting site
Florian	Losch		MD	Vivantes Humboldt Hospital Berlin, Department of Neurology	Berlin, Germany	Reporting Physician	Local recruiting site
Wenke	Grönheit		MD	Centre for Neurology and Epilepsy, Knappschaft-Hospital, Bochum-Langendreer	Bochum, Germany	Reporting Physician	Local recruiting site
Matthias	Lindenau		MD	Centre for Neurology, Private Practice "Neurologie Neuer Wall, Dr. Bredow & Partner"	Hamburg, Germany	Reporting Physician	Local recruiting site
Ramshekhar	Menon		MD, PhD	Department of Neurology, SCTIMST	Trivandrum, India	Reporting Physician	Local recruiting site
Jafar	Mehvari Habibabadi		Professor,	Isfahan University of medical sciences	Isfahan, Iran	Reporting Physician	Local recruiting site
Maria P.	Canevini		MD	Università degli Studi di Milano	Milan, Italy	Reporting Physician	Local recruiting site

Supplemental Online Content: Nonauthor Collaborators
*First name, last name, and suffix (if applicable) are required and will appear in PubMed.

***First Name and Middle Initial(s)**	***Last Name**	***Suffix (eg, Jr, III)**	Academic Degrees	Institution	Location (city, state/province, country)	Role or Contribution, eg, chair, principal investigator	Group (if more than 1 Group listed in the byline) and/or Subgroup (eg, Steering Committee)
Elena	Zambrelli		MD	Childhood and Adolescence Neuropsychiatry Unit, ASST Santi Paolo e Carlo, San Paolo Hospital	Milan, Italy	Reporting Physician	Local recruiting site
Katherine	Turner		PsyD	Childhood and Adolescence Neuropsychiatry Unit, ASST Santi Paolo e Carlo, San Paolo Hospital	Milan, Italy	Reporting Physician	Local recruiting site
Michela	Cecconi		MD	complex structure of neurophysiopathology Azienda Ospedaliera di Perugia	Perugia, Italy	Reporting Physician	Local recruiting site
Aldo	Paggi		MD	Private Neurologist	Ancona, Italy	Reporting Physician	Local recruiting site
Nicoletta	Foschi		MD	Ospedale Regionale Torrette	Ancona, Italy	Reporting Physician	Local recruiting site
Antonio	Gambardella		Professor,	University Magna Graecia	Catanzaro, Italy	Reporting Physician	Local recruiting site
Simone	Beretta		MD, PhD	Fondazione IRCCS San Gerardo Monza, University of Milano-Bicocca	Monza, Italy	Reporting Physician	Local recruiting site
Angela	Giglio		MD	Fondazione IRCCS San Gerardo Monza, University of Milano-Bicocca	Monza, Italy	Reporting Physician	Local recruiting site
Gaia	Fanella		MD	Fondazione IRCCS San Gerardo Monza, University of Milano-Bicocca	Monza, Italy	Reporting Physician	Local recruiting site
Lorenzo	Ferri		MD	IRCCS Istituto delle Scienze Neurologiche di Bologna- Full Member of the ERN EpiCARE	Bologna, Italy	Reporting Physician	Local recruiting site
Francesca	Bisulli		MD, PhD	IRCCS Istituto delle Scienze Neurologiche di Bologna- Full Member of the ERN EpiCARE & Department of Biomedical and Neuromotor Sciences, University of Bologna	Bologna, Italy	Reporting Physician	Local recruiting site
Alessandra	Pistelli		MD, PhD	Centro di Riferimento Regionale di Tossicologia Perinatale, S.O.D.c Tossicologia Medica, Azienda Ospedaliero Universitaria Careggi	Firenze, Italy	Reporting Physician	Local recruiting site

*First name, last name, and suffix (if applicable) are required and will appear in PubMed.

***First Name and Middle Initial(s)**	***Last Name**	***Suffix (eg, Jr, III)**	Academic Degrees	Institution	Location (city, state/province, country)	Role or Contribution, eg, chair, principal investigator	Group (if more than 1 Group listed in the byline) and/or Subgroup (eg, Steering Committee)
Pietro	Pignatta		MD	Humanitas Gradenigo	Turin, Italy	Reporting Physician	Local recruiting site
Marta	Maschio		MD	Center for Tumor-related Epilepsy, UOSD Neuroncology IRCCS “Regina Elena” National Cancer Institute	Rome, Italy	Reporting Physician	Local recruiting site
Francesca	Muzzi		MD, PhD	Centro Epilessia LICE I Livello Medico Adulti ASL ROMA 3	Roma, Italy	Reporting Physician	Local recruiting site
Maria S.	Cotelli		MD	ASST Valcamonica	Esine, Italy	Reporting Physician	Local recruiting site
Etsuko	Yamazaki		MD, PhD	Shizuoka Institute of Epilepsy and Neurological Disorders	Shizuoka, Japan	Reporting Physician	Local recruiting site
Kiyohito	Terada		MD, PhD	Shizuoka Institute of Epilepsy and Neurological Disorders	Shizuoka, Japan	Reporting Physician	Local recruiting site
Yushi	Inoue		MD, PhD	Shizuoka Institute of Epilepsy and Neurological Disorders	Shizuoka, Japan	Reporting Physician	Local recruiting site
Masahiro	Mizobuchi		MD, PhD	Nakamura-Kinen Hospital	Sappolo, Japan	Reporting Physician	Local recruiting site
Katsuyuki	Fukushima		MD, PhD	Fukushima Neurology Clinic	Hokkaido, Japan	Reporting Physician	Local recruiting site
Masaaki	Kato		MD, PhD	MusashinoKokubunnzi Clinic	Tokyo, Japan	Reporting Physician	Local recruiting site
Takahiro	Mitsueda		MD, PhD	Japanese Red Cross Otsu Hospital	Otsu, Japan	Reporting Physician	Local recruiting site
Torleiv	Svendsen		MD, PhD	The National Center for Epilepsy, Member of the ERN EpiCARE, Oslo University Hospital, Oslo; Innlandet Hospital Trust, Lillehammer	Lillehammer & Oslo, Norway	Reporting Physician	Local recruiting site
Erik	Taubøll		MD, PhD, F	Department of Neurology, Oslo University Hospital, Oslo, Institute of Clinical Medicine, University of Oslo, Oslo	Oslo, Norway	Reporting Physician	Local recruiting site
Alma	Sikiric		MD	Department of Neurohabilitation, Oslo University Hospital, Oslo	Ulleval, Oslo, Norway	Reporting Physician	Local recruiting site
Katrine	Haggag		MD	Department of Neurohabilitation, Oslo University Hospital, Oslo	Ulleval, Oslo, Norway	Reporting Physician	Local recruiting site
Eline	Dahl-Hansen		MD	Department of Neurology, Drammen Hospital, Vestre Viken HF, Drammen	Drammen, Norway	Reporting Physician	Local recruiting site

*First name, last name, and suffix (if applicable) are required and will appear in PubMed.

*First Name and Middle Initial(s)	*Last Name	*Suffix (eg, Jr, III)	Academic Degrees	Institution	Location (city, state/province, country)	Role or Contribution, eg, chair, principal investigator	Group (if more than 1 Group listed in the byline) and/or Subgroup (eg, Steering Committee)
Ineke	Hogenesch		MD	Department of Neurology, Helse Fonna, Haugesund	Haugesund, Norway	Reporting Physician	Local recruiting site
Eylert	Brodtkorb		MD, PhD, F	Department of Neurology and Clinical Neurophysiology, St.Olav University Hospital, Trondheim; Department of Neuromedicine and Movement Science, Norwegian University of Science and Technology, Trondheim	Trondheim, Norway	Reporting Physician	Local recruiting site
Isabel	Pires		MD	Neurophysiology Unit, Department of Neurology, Centro Hospitalar Universitário de São João	Porto, Portugal	Reporting Physician	Local recruiting site
Helena	Rocha		MD	Neurophysiology Unit, Department of Neurology, Centro Hospitalar Universitário de São João	Porto, Portugal	Reporting Physician	Local recruiting site
Marta	Carvalho		MD	Neurophysiology Unit, Department of Neurology, Centro Hospitalar Universitário de São João	Porto, Portugal	Reporting Physician	Local recruiting site
Ricardo	Rego		MD	Neurophysiology Unit, Department of Neurology, Centro Hospitalar Universitário de São João	Porto, Portugal	Reporting Physician	Local recruiting site
Carla	Bentes		MD, PhD	Centro de Referência Epilepsias Refratárias (EpiCARE member), Centro Hospitalar Universitário Lisboa Norte & Centro de Estudos Egas Moniz, Faculdade de Medicina da Universidade de Lisboa	Lisboa, Portugal	Reporting Physician	Local recruiting site

Supplemental Online Content: Nonauthor Collaborators
*First name, last name, and suffix (if applicable) are required and will appear in PubMed.

*First Name and Middle Initial(s)	*Last Name	*Suffix (eg, Jr, III)	Academic Degrees	Institution	Location (city, state/province, country)	Role or Contribution, eg, chair, principal investigator	Group (if more than 1 Group listed in the byline) and/or Subgroup (eg, Steering Committee)
Ana C.	Gonçalves Franco		MD	Centro de Referência Epilepsias Refratárias (EpiCARE member), Centro Hospitalar Universitário Lisboa Norte & Centro de Estudos Egas Moniz, Faculdade de Medicina da Universidade de Lisboa	Lisboa Portugal	Reporting Physician	Local recruiting site
Sara	Parreira		MD	Centro de Referência Epilepsias Refratárias (EpiCARE member), Centro Hospitalar Universitário Lisboa Norte & Centro de Estudos Egas Moniz, Faculdade de Medicina da Universidade de Lisboa	Lisboa, Portugal	Reporting Physician	Local recruiting site
Halina	Navumava		MD	Center of paroxysmal conditions, Vicebsk Regional Diagnostic Center	Vicebsk, Republic of Belarus	Reporting Physician	Local recruiting site
Ksenija	Gebauer Bukurov		Professor,	Faculty of Medicine, University of Novi Sad & Clinic of Neurology, University Clinical Centre of Vojvodina	Novi Sad, Serbia	Reporting Physician	Local recruiting site
Juan L.	Becerra Cuñat		MD	Hospital Universitari Germans Trias i Pujol	Badalona, Spain	Reporting Physician	Local recruiting site
Clara	Cabeza Alvarez		MD	Hospital Universitario de Toledo	Toledo, Spain	Reporting Physician	Local recruiting site
Mercedes	Garces Sanchez		MD	Hospital Universitari i Politèctic la Fe	Valencia, Spain	Reporting Physician	Local recruiting site
Gemma	Sansa Fayos		MD	Hospital Universitari Parc Tauli	Sabadell, Spain	Reporting Physician	Local recruiting site
David	Sopelana Garay		MD	Hospital General Universitario de Albacete	Albacete, Spain	Reporting Physician	Local recruiting site
Alvaro	Sanchez Larsen		MD	Hospital General Universitario de Albacete	Albacete, Spain	Reporting Physician	Local recruiting site
Maria D.	Castro Vilanova		MD	Alvaro Cunquerio University Hospital	Vigo, Spain	Reporting Physician	Local recruiting site
Lisa	Gordon		MD	Karolinska University Hospital	Stockholm, Sweden	Reporting Physician	Local recruiting site
Peter	Mattsson		MD, PhD, A	Dept.of Medical Sciences, Neurology	Uppsala, Sweden	Reporting Physician	Local recruiting site

Supplemental Online Content: Nonauthor Collaborators
*First name, last name, and suffix (if applicable) are required and will appear in PubMed.

***First Name and Middle Initial(s)**	***Last Name**	***Suffix (eg, Jr, III)**	Academic Degrees	Institution	Location (city, state/province, country)	Role or Contribution, eg, chair, principal investigator	Group (if more than 1 Group listed in the byline) and/or Subgroup (eg, Steering Committee)
Eva	Kumlien		MD, PhD, A	Dept.of Medical Sciences, Neurology	Uppsala, Sweden	Reporting Physician	Local recruiting site
Maria	Strandberg		MD, PhD	Division of Neurology, Department of Clinical Sciences, Lund University Hospital	Lund, Sweden	Reporting Physician	Local recruiting site
Avan S.	Rashid		MD	Department of Neurology, and Department of Biomedical and Clinical Sciences, Linköping University	Linköping, Sweden	Reporting Physician	Local recruiting site
Helena	Gauffin		MD, PhD	Department of Neurology, and Department of Biomedical and Clinical Sciences, Linköping University	Linköping, Sweden	Reporting Physician	Local recruiting site
Irene	Hakansson		MD, PhD	Department of Neurology, and Department of Biomedical and Clinical Sciences, Linköping University	Linköping, Sweden	Reporting Physician	Local recruiting site
Maria	Bograkou		MD	Department of Neurology, and Department of Biomedical and Clinical Sciences, Linköping University	Linköping, Sweden	Reporting Physician	Local recruiting site
Kristina	Malmgren		Professor,	Department of Clinical Neuroscience, Institute of Neuroscience and Physiology, Sahlgrenska Academy, University of Gothenburg	Göteborg, Sweden	Reporting Physician	Local recruiting site
Dominique	Flügel		MD	Department of Neurology, Cantonal Hospital St Gallen	St Gallen, Switzerland	Reporting Physician	Local recruiting site
Stephan	Rüegg		Professor, MD, PhD, FEAN, FAES	Department of Neurology, University Hospital Basel	Basel, Switzerland	Reporting Physician	Local recruiting site
Martin	Kurthen		Professor,	Swiss Epilepsy Center - Klinik Lengg	Zurich, Switzerland	Reporting Physician	Local recruiting site

Supplemental Online Content: Nonauthor Collaborators
*First name, last name, and suffix (if applicable) are required and will appear in PubMed.

***First Name and Middle Initial(s)**	***Last Name**	***Suffix (eg, Jr, III)**	Academic Degrees	Institution	Location (city, state/province, country)	Role or Contribution, eg, chair, principal investigator	Group (if more than 1 Group listed in the byline) and/or Subgroup (eg, Steering Committee)
Dilek	Atakli		MD, PhD	Istanbul Bakırköy Training and Research Hospital for Psychiatry, Neurology and Neurosurgery	Bakırköy, İstanbul, Turkey	Reporting Physician	Local recruiting site
Önder	Kemal Soylu		MD	Istanbul Bakırköy Training and Research Hospital for Psychiatry, Neurology and Neurosurgery	Bakırköy, İstanbul, Turkey	Reporting Physician	Local recruiting site
Janet E	Graham			Australian Pregnancy Register, Royal Melbourne Hospital	Parkville, Australia	Study Coordinator	Local recruiting site
Alison A	Hitchcock			Australian Pregnancy Register, Royal Melbourne Hospital	Parkville, Australia	Study Coordinator	Local recruiting site
Stefanie	Gadeyne			Ghent University Hospital	Ghent, Belgium	Study Coordinator	Local recruiting site
Katherine	Moreno			Liga Chilena contra la Epilepsia	Santiago, Chile	Study nurse	Local recruiting site
Hanna	Jersing			Rigshospitalet, Copenhagen University Hospital	Copenhagen, Denmark	Study nurse	Local recruiting site
Karina	Als			University Hospital Aalborg	Aalborg, Denmark	Study nurse	Local recruiting site
Lone	Olsen			The Danish Epilepsy center, Filadelfia	Dianalund, Denmark	Study nurse	Local recruiting site
Julie	Althoen Sønderup			Aarhus Universitetshospital, Palle Juul-Jensens Bouldevard 165	Aarhus N, Denmark	Study Coordinator	Local recruiting site
Pirjo	Lavi			Kuopio University Hospital	Kuopio, Finland	Study Coordinator	Local recruiting site
Kirsten	Krämer			Vivantes Humboldt Hospital Berlin, Department of Neurology	Berlin, Germany	Study nurse	Local recruiting site
Siri	Myklebust			The National Center for Epilepsy, Member of the ERN EpiCARE, Oslo University Hospital, Oslo	Oslo, Norway	Study nurse	Local recruiting site
Mariann	Høgli Grøtte			The National Center for Epilepsy, Member of the ERN EpiCARE, Oslo University Hospital, Oslo	Oslo, Norway	Study nurse	Local recruiting site
Bibiane	Moche Kamga			Fondazione IRCCS Istituto Neurologico Carlo Besta	Milan, Italy	Registry Data Manager and Supervisor	EURAP Central Registry
Laura	Gargantini			Fondazione IRCCS Istituto Neurologico Carlo Besta	Milan, Italy	Registry Auditor	EURAP Central Registry
Cristina	Bonato			Fondazione IRCCS Istituto Neurologico Carlo Besta	Milan, Italy	Registry Auditor	EURAP Central Registry

Supplemental Online Content: Nonauthor Collaborators
*First name, last name, and suffix (if applicable) are required and will appear in PubMed.

***First Name and Middle Initial(s)**	***Last Name**	***Suffix (eg, Jr, III)**	Academic Degrees	Institution	Location (city, state/province, country)	Role or Contribution, eg, chair, principal investigator	Group (if more than 1 Group listed in the byline) and/or Subgroup (eg, Steering Committee)
Stefania	Falchi			Fondazione IRCCS Istituto Neurologico Carlo Besta	Milan, Italy	Registry secretary	EURAP Central Registry
