# Supplementary material for: Risk of Major Congenital Malformations and Exposure to Antiseizure Medication Monotherapy
Source: JAMA Neurol. 2024 Mar 18;81(5):481–9. doi: 10.1001/jamaneurol.2024.0258 (PMC10949148; doi:10.1001/jamaneurol.2024.0258)
Supplement: Supplement 3. — Data Sharing Statement. [file jamaneurol-e240258-s003.pdf]

Data Sharing Statement

Battino. Risk of Major Congenital Malformations and Exposure to Antiseizure Medication Monotherapy. *JAMA Neurol*. Published March 18, 2024. doi:10.1001/jamaneurol.2024.0258

Data
Data available: No
